# Use of a group concept mapping approach to define learning outcomes for an interdisciplinary module in medicine

**DOI:** 10.1007/s40037-013-0095-7

**Published:** 2013-12-10

**Authors:** Slavi Stoyanov, Howard Spoelstra, Deirdre Bennett, Catherine Sweeney, Sabine Van Huffel, George Shorten, Siun O’Flynn, Padraig Cantillon-Murphy, Colm O’Tuathaigh, Louise Burgoyne

**Affiliations:** 1 Centre for Learning Sciences and Technologies, Open Universiteit Nederland, Valkenburgerweg 177, 6419 AT Heerlen, the Netherlands; 2 School of Medicine, Brookfield Heatlh Sciences Complex, University College Cork, Cork, Ireland; 3 Department of Electrical Engineering (ESAT-SCD) and iMinds Future Health Department, Katholieke Universiteit Leuven, 3001 Louvain, Belgium; 4 School of Engineering, University College Cork, College Road, Cork, Ireland

**Keywords:** Group concept mapping, Interdisciplinary learning, Medicine

## Abstract

Learning outcomes are typically developed using standard group-based consensus methods. Two main constraints with standard techniques such as the Delphi method or expert working group processes are: (1) the ability to generate a comprehensive set of outcomes and (2) the capacity to reach agreement on them. We describe the first application of Group Concept Mapping (GCM) to the development of learning outcomes for an interdisciplinary module in medicine and engineering. The biomedical design module facilitates undergraduate participation in clinician-mentored team-based projects that prepare students for a multidisciplinary work environment. GCM attempts to mitigate the weaknesses of other consensus methods by excluding pre-determined classification schemes and inter-coder discussion, and by requiring just one round of data structuring. Academic members from medicine and engineering schools at three EU higher education institutions participated in this study. Data analysis, which included multidimensional scaling and hierarchical cluster analysis, identified two main categories of outcomes: technical skills (new advancement in design process with special attention to users, commercialization and standardization) and transversal skills such as working effectively in teams and creative problem solving. The study emphasizes the need to address the highest order of learning taxonomy (analysis, synthesis, problem solving, creativity) when defining learning outcomes.

## Introduction

Clinician-mentored biomedical device design modules are well established and have a proven track record of commercial project outputs and follow-on research [1, 2]. Undergraduate medical and engineering students enrolled in the interdisciplinary biomedical design module at University College Cork (UCC, Ireland) are taught biomedical device design-targeted knowledge and problem solving skills, and participate in clinician-mentored team-based projects that prepare them for today’s multidisciplinary work environment [3].

Learning outcomes describe what a learner is expected to know, understand and be able to do after successful completion of a process of learning [4]. Learning outcomes (LOs) are part of an international move away from traditional university teaching methods which focused primarily on the student’s ability to absorb knowledge. Outcome-based teaching and learning focuses on the equally if not more important student ability to put knowledge to use in solving problems, and operating effectively in a chosen field [5]. Development of LOs is now standard practice in higher education. Various methods are used to facilitate definition of specific module LOs from experts and students such as survey-based questionnaires [6], the Delphi method [7], student self-assessments [8] and expert working groups [9, 10].

Constraints associated with standard means of reaching group consensus with respect to defining LOs are: (a) generating a *comprehensive* set of LOs, and (b) reaching *agreement* on them. Agreement on LOs (and how much emphasis should be placed on each one) may be even more difficult to achieve when participants represent different professional domains such as medicine and engineering.

One solution to the issues just mentioned is to use group concept mapping (GCM) [11, 12]. This research methodology, while building on the strengths of other structured consensus building methods such as focus groups and the Delphi method, mitigates some of their weaknesses. In contrast to the Delphi method, in GCM there is only one round of data structuring as the participants work independently and anonymously of each other to limit the possibility of ‘groupthink’ or ‘peer pressure’. Unlike interviews and focus groups, GCM does not rely on pre-determined classification schemas. The method does not need inter-coder discussion to come up with an agreement. When sorting the statements into groups, the participants, in fact, ‘code’ the text themselves. Multivariate statistical analysis then aggregates the individual coding schemas across the participants. Consensus is not forced, but emerges organically from the data.

This paper describes the first application of GCM towards generation of LOs for an interdisciplinary module in medicine and engineering.

## Methods

The project consortium was composed of academic members from medicine and engineering schools at three EU higher education institutions (University College Cork, Ireland; Open Universiteit Nederland, Netherlands; and Katholieke Universiteit Leuven, Belgium). The GCM procedure consisted of five phases: (1) idea generation (brainstorm) and idea pruning, (2) sorting of ideas into groups, (3) rating on two values (‘importance’ and ‘difficulty to achieve’), (4) analysis of the data and (5) interpretation of the results. Project members were provided with a web-based link to a web-based tool for data collection and analysis (Concept System Global 2012). They were asked to generate ideas completing the following trigger statement: ‘One specific learning outcome of the Biomedical Design module is…….’.

The resulting list of ideas was then made available to a smaller expert group comprising project members from each discipline and partner institution, firstly for the sorting of ideas into categories (based on similarity in meaning), giving names to the categories, and secondly for the rating of the ideas on two values—*importance to achieve* and *difficulty to achieve*. Data analysis included multidimensional scaling and hierarchical cluster analysis (HCA) for sorting of data, and means, standard deviations, and correlations for rating of data.

## Results

Nineteen experts from the consortium responded positively to the invitation to participate in the study. Sixteen members contributed to the idea-generation phase, nine to the sorting phase, and seven to the rating phase.

### Clustering results

The first step in the GCM data analysis is clustering. An important representation validity estimate here is stress value. It reflects the goodness-of-fit, i.e. how accurately the concept map represents the way the participants structured and organized the information. The stress value of this GCM study was 0.28, which is the same as the average stress value reported in a meta-analytical study of 69 GCM projects [13]. Clustering uses multidimensional scaling (MDS; assigning each statement a bridging value, which is between 0 and 1) to position the learning outcome statements. HCA was employed to cluster groups of LOs. Several clustering solutions were checked, applying the practical heuristic’ 20-to-5′. This means we started from a 20-cluster solution with the goal to arrive at a 5-cluster solution.

The next step was to attach meaningful labels to the clusters. There are three GCM methods available for labelling. The first method is to check what the system suggests. This means that the system suggests a label for a group of statements, based on the label given by a group member whose centroid is the closest to the centroid of the cluster formed by the aggregation of the data from all the members. The second method is to look at the bridging values of the statements composing the cluster. The statements with lower bridging values typically represent a cluster better. The third method is to read through all the statements in a cluster and to define in a label what the story is behind the learning outcome statements (what does the cluster want to tell us?). To define the cluster labels for this study (e.g., collective theme of the statements, or category) we combined all three methods. The following clusters were identified:

1. *Attention to end user* is about the need to take the characteristics of biomedical design end users (doctors, nurses, patients) into account.
2. *New approaches* emphasizes the need to look for new design methodologies, include results from design research, and implement original ideas in designing medical devices.
3. *Design process* refers to knowledge and skills related to conducting high-quality design activities, from need assessment to developing and testing working prototypes.
4. *Regulation and ethics* focuses on the need to be aware about regulations, standards, quality controls and ethical norms when designing medical devices.
5. *Commercialization* suggests considering possibilities for entering the market, and related knowledge and skills for making the product commercially attractive.
6. *Knowledge integration* highlights the need for combining knowledge and research from different professional domains.
7. *Communication*, as the name suggests, is about having the skills to communicate effectively with representatives from other professional domains.
8. *Collaboration* includes a range of ideas from specific issues of creative team dynamics.
9. *Higher order skills* suggests focusing on the highest level of learning taxonomy: creative problem solving, experimentation, analysis and synthesis.
10. *Problem solving process* is about effective and efficient problem solving skills (analysis of problem situation, idea generation, applying new problem solving methodologies, and awareness of own and others problem solving styles).
11. *Connecting domains* is about recognizing and evaluating connections to different concepts, fields and contexts.
12. *Learning goals* lists a number of learning goals and some more specific learning objectives.

The average bridging value among the clusters was 0.37. High coherence within a cluster (lowest bridging values) means that the most people agreed on the LOs. This applies to the clusters ‘higher order skills’ (0.03), followed by ‘learning goals’ (0.10), ‘problem solving process’ (0.18) and connecting domains (0.20). The clusters with the highest bridging values were ‘regulation and ethics’ (0.71) and commercialization’ (0.70).

### Rating results

**Fig. 1:**
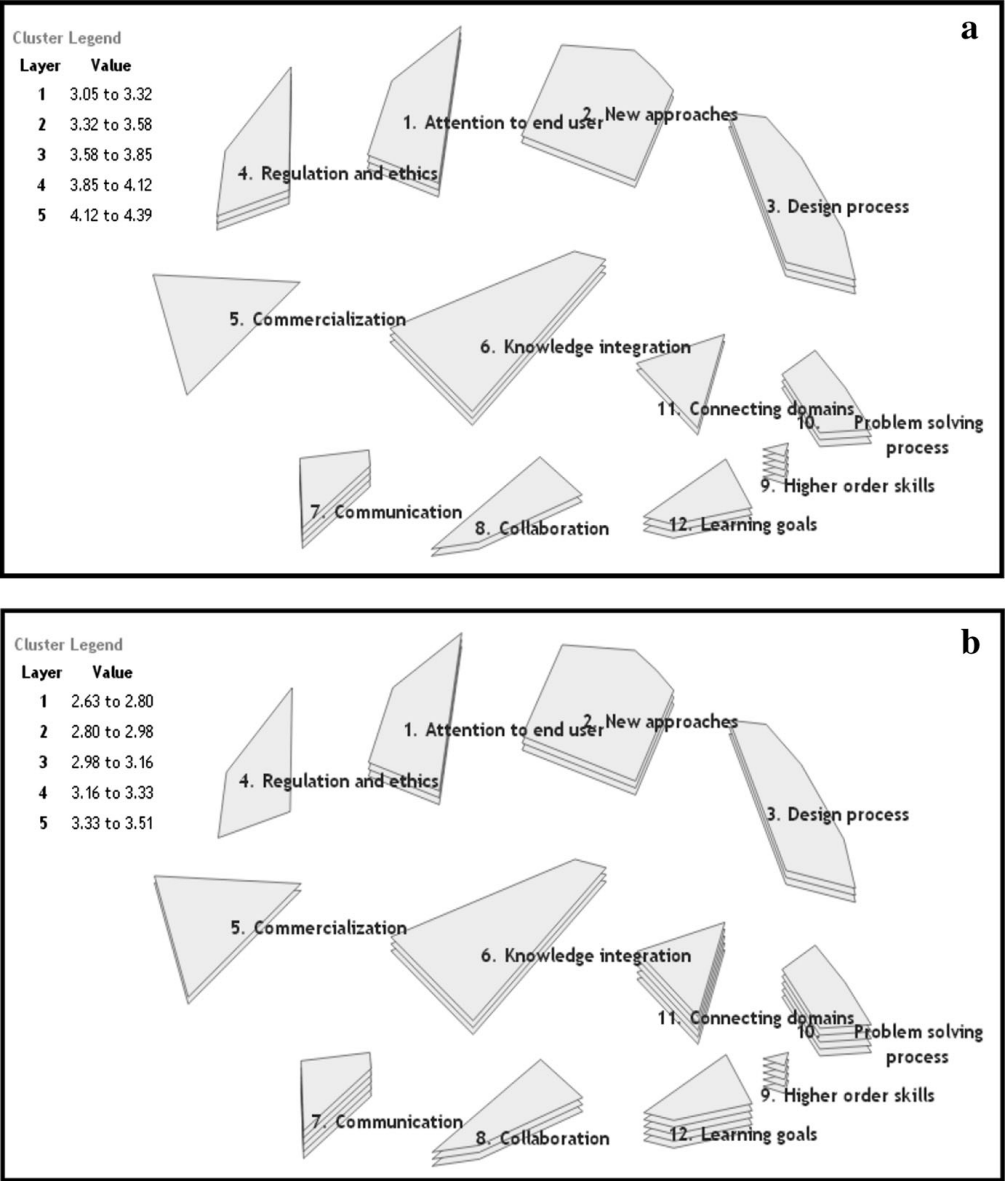
**a** Ratings of GCM clusters on ‘importance to achieve’ *Layer* how important are the learning outcomes from 1 ‘not at all important’ to 5 ‘very important’. *Value* cluster mean range. **b** Ratings of GCM clusters on ‘difficulty to achieve’. *Layer* how difficult is it to achieve the learning outcomes from 1 ‘not at all difficult’ to 5 ‘very difficult’. *Value* cluster mean range

The GCM system provides a visualization of the expert group rating results. High rating results are depicted as high numbers of cluster layers. Figure 1a, b depict the layers representing the rating category outcomes ‘Importance to achieve’ and ‘Difficulty to achieve’. Pearson’s product-moment correlation coefficient test showed a moderate correlation between both measures (r = 0.52, *p* < 0.001).

## Discussion

The GCM analysis shows that ‘higher order skills’ and ‘communication’ are the two most important clusters of interdisciplinary LOs. However, these outcomes also require the most effort before they can be achieved. The clusters ‘learning goals’, ‘problem solving process’ and ‘connecting domains’ also contain outcomes that are perceived by the group to be difficult to achieve. The least important cluster from the expert group analysis was ‘commercialization’ and the cluster ‘regulation and ethics’ was considered to contain outcomes that were easy to achieve.

This GCM framework reveals not only LOs related to traditional topics such as ‘design process’ and ‘creative problem solving’, but also draws attention to educational outcome themes such as ‘commercialization’, ‘standardization’, ‘regulations’, and ‘ethics’. The results suggest emphasizing elements of the highest levels in learning taxonomies by defining LOs such as ‘analysis’, ‘synthesis’, ‘problem solving’ and ‘combining knowledge from different professional domains’ for either informing the design process (e.g., implementing recent developments in software design to the biomedical design process) or stimulating creativity (e.g. creative strategies of ‘looking in other worlds’, ‘making novel combinations’ and ‘connecting the unconnected’).

### Validity and reliability considerations

High values for GCM on internal validity and reliability estimates were reported in a recent meta-analysis, where quality and rigour of GCM was compared with other mixed participative methodologies [13]. One important validity criteria is how well the mathematical model produced by multidimensional analysis (MDS) and HCA reflects the judgements of the participants in the study. To estimate an acceptable level on the stress index in GCM studies, the authors of the meta-analysis refer to a simulated study [14]. Here 500,000 matrices were calculated with 100 objects scaled. Results showed that for two-dimensional multidimensional (MDS) solutions where 100 objects have been scaled, there is a 1 % chance the arrangement of the objects in the matrix is random if the stress value is below an upper limit of 0.39. The stress value of 0.28 for this study is considered a very good estimate for the internal representation validity of this study given the small sample size.

Criteria for external validity of GCM studies require involvement of independent experts with different perspectives on the issues at hand, a variety of methods for data collection, and the extent to which the brainstormed statements reflect the reality as constructs and scope. Our study included experts with educational backgrounds in engineering and computer science, medicine and healthcare, social sciences, maths and science and business and management. In the instruction for idea generation, we explicitly advised the participants to brainstorm ideas and also to refer to information in dedicated written sources—journals, books, reports, blogs or personal communication with other experts. The final list contained 86 ideas. We are not aware of any other study on LOs that has produced such a large number of ideas. When presented with the final cluster solution, our participants acknowledged that the list of ideas was comprehensive, that the concept map reflected the way they had structured the information and that the analysis generated an even richer picture than they were expecting.

In the present study, the number of contributing consortium group members in the sorting and rating activities was low. However, given the acceptable study stress value of 0.28 and considering that studies in other professional domains (usability) [15] claim that there is a 0.75 correlation between the results from five participants and ultimate results, we believe that multidimensional scaling using the sorting data from nine people produced an accurate picture.

## Conclusions

Our GCM study identified content areas related to biomedical design module LOs which can be grouped into two main categories: technical skills (new advancements in design process with special attention to users, commercialization and standardization) and transversal skills such as working effectively in teams and creative problem solving.

This study provides not only an empirical basis for depicting the main learning outcome areas, but also suggests how to operationally define them (through the statements in each cluster). It emphasizes the need to address the highest level of learning taxonomy (analysis, synthesis, problem solving, creativity) when defining LOs. The same methodology can be applied to address various issues related to medical education: from defining LOs within different medical discipline modules to classifying different teaching techniques. The GCM method is particularly useful for defining LOs within the growing number of interdisciplinary modules offered at undergraduate level, stemming from recommendations to foster transferable skills such as communication, teamwork, time management, critical thinking, and research specific skills [16–18].

## References

[1] Hanumara M, Walsh C, Osborn L, Slocum A. Solving medical challenges while teaching mechanical engineering design. ASME J Mech Des, 2013 (in press).

[2] Bright A, Phillips JR (1999). The harvey mudd engineering clinic: past present and future. Int J Eng Educ..

[3] Cantillon-Murphy P, McSweeney J, Burgoyne L, O’Tuathaigh C, O’Flynn S. Solving clinical problems through interdisciplinary learning: an initial feasibility study. Int J Eng Educ. 2013 (in press).

[4] ECTS Users’ Guide (2009), Brussels: Directorate-General for Education and Culture. http://ec.europa.eu/education/lifelong-learning-policy/doc/ects/guide_en.pdf.

[5] Biggs J, Tang C (2009) Applying constructive alignment to outcomes based teaching and learning. http://drjj.uitm.edu.my/DRJJ/MQAGGPAS-Apr2011/What-is-CA-biggs-tang.pdf.

[6] Kordi R, Dennick RG, Scammell BE (2005). Developing learning outcomes for an ideal MSc course in sports and exercise medicine. Br J Sports Med.

[7] Tonni I, Oliver R (2013). A Delphi approach to define learning outcomes and assessment. Eur J Dent Educ.

[8] Schiekirka S, Reinhardt D, Beibbarth T, Anders S, Pukrop T, Raupach T (2013). Estimating learning outcomes from pre- and posttest student self-assessments: a longitudinal study. Acad Med.

[9] Johnson O, Bailey ST, Willcott C (2012). Global health learning outcomes for medical students in the UK. Lancet.

[10] Burke S, Martin M, Thomas H, Farndon P (2009). The development of core learning outcomes relevant to clinical practice: identifying priority areas for genetics education for non-genetics specialist registrars. Clin Med.

[11] Trochim WMK (1989). An introduction to concept mapping for planning and evaluation. Eval Program Plann..

[12] Trochim MK, Trochim WMK (2007). Concept mapping for planning and evaluation. Applied social research methods series.

[13] Rosas SR, Kane M (2012). Quality and rigor of the concept mapping methodology: a pooled study analysis. Eval Program Plann..

[14] Sturrock K, Rocha J (2000). A multidimensional scaling stress evaluation table. Field Methods..

[15] Turner CW, Lewis JR, Nielsen J, Karwowski (2006). Determining usability test sample size. International encyclopedia of ergonomics and human factors.

[16] General Medical Council UK. Tomorrows Doctors, outcomes and standards for undergraduate medical education; 2009.

[17] Scottish Deans Medical Education Group. 3. Edinburgh, UK: Scottish Deans Medical Education Group; 2009. The Scottish Doctor: learning outcomes for the medical undergraduate in Scotland: a foundation for competent and reflective practitioners. 10.1080/01421590220120713 12098432

[18] National Alliance for Physician Competence. Good medical practice—USA: the National Alliance. Version 1.0. 2007. http://www.gmpusa.org.

